# Of Functional Nervous Disease

**Published:** 1915-09-18

**Authors:** 


### OF FUNCTIONAL NERVOUS DISEASE.

THE division of diseases into structural and functional groups, however convenient for practical purposes, has never been judged quite satisfactory by medical authorities. It is, however, not easy to find an effective substitute for this classification. The case may be argued as an academic exercise that where there is disturbance of function there must necessarily be anatomical change, and, as a general proposition, it may be impossible to contest this. Yet experience teaches that disease in the shape of symptoms may exist even when the most scrupulous search by all known methods of investigation fails to find any clinical evidence of modified structure; and the complete disappearance of such symptoms—possibly with dramatic suddenness—appears to confirm the negative conclusion. In some instances of this order it may be possible to suggest the presence in the body of some poisonous or other disturbing agency as the cause of the symptoms, and the elimination of such agency as the explanation of the cure. But there are many other experiences where this proposition is difficult or impossible. The mental and emotional strains suffered by men exposed to the horrors of the trenches are just now making some of these experiences conspicuous, and some " wonderful cures " offer tempting opportunities for the lay Press.

The medical profession may be puzzled about the exact nature of the underlying disturbance in so-called functional nervous disease, but the condition called by this name is, of course, familiar to every practitioner. The same remark may be made about the sudden disappearance of the symptoms, perhaps under some exciting or emotional influence. One of the practical difficulties of the situation created by such events is to prevent the person principally concerned from becoming the subject of serious misconstruction. The change is conspicuous, and is seen and known of all men. The man who was blind now sees, or the dumb man speaks, or the paralysed resumes his activities. The tendency in one direction is to record amazement at the wonderful or even miraculous cure, while from another side comes the suggestion that the patient must have been malingering or shamming. In the one case he is the nine days' wonder of his friends and of the local Press; in the other he is the victim of unpleasant suspicions.

There is no difficulty in saying that in the cases now in question neither the one nor the other of the suggestions just indicated is justified. It may not always be easy to separate these so-called functional cases from cases of malingering, but, with rare exceptions, it is possible to affirm the presence or absence of evidence of structural change. Hence the sudden return of sight or of the power of speech when no manifest alteration in the corresponding organs can be appreciated is never some extraordinary therapeutic achievement, but is in strict harmony with a well-established experience. The event may be hard to explain as a physiological happening, and even the psychologists may be unable to penetrate to the heart of it. But to sound it abroad as a wonderful cure is merely absurd. On the other side, substantial injustice may be done to individuals by a failure on the part of friends to realise that symptoms may be genuine even when no organic interpretation of them can be discovered. There is need to press this statement just now, as cases of this order are being observed in all classes of the community, and not infrequently they are being misinterpreted in one or other of the two directions here suggested. That there are malingerers is quite true, and the detection of these is an achievement of native ingenuity rather than of special medical skill. But much injustice will be done if there is a failure to recognise that though, equally with the malingerer, the victim of functional nervous disease shows no evidence of organic change, the latter is none the less a genuine sufferer.

